# Association of concomitant laparoscopy with reproductive outcomes after hysteroscopic septum resection: a longitudinal cohort study

**DOI:** 10.3389/frph.2026.1950477

**Published:** 2026-09-18

**Authors:** Yu Lu, Mi Su, Qinqin Yuan, Li He

**Affiliations:** Department of Gynecology, Chengdu Women’s and Children’s Central Hospital, School of Medicine, University of Electronic Science and Technology of China, Chengdu, China

**Keywords:** ESHRE/ESGE classification, hysteroscopy, laparoscopy, live birth, reproductive outcomes, uterine septum

## Abstract

**Objective:**

To evaluate whether concomitant laparoscopy during hysteroscopic uterine septum resection is associated with perioperative characteristics and postoperative reproductive outcomes, and to explore the prognostic roles of ESHRE/ESGE classification and septum depth.

**Methods:**

This single-center retrospective cohort study included 361 patients who underwent hysteroscopic septum resection between 2017 and 2024. Of these, 241 underwent hysteroscopy alone and 120 underwent concomitant laparoscopy. Multivariable logistic regression was used to evaluate postoperative pregnancy, live birth, and miscarriage. Kaplan–Meier analysis and Cox regression were used to assess time to first postoperative pregnancy. Anatomical analyses included U2a/U2b classification and septum depth.

**Results:**

Postoperative pregnancy rates were 56.8% and 60.8% in the hysteroscopy-only and concomitant-laparoscopy groups, respectively (*P* = 0.469), while live-birth rates were 42.7% and 39.2% (*P* = 0.517). After adjustment for clinical characteristics and ESHRE/ESGE classification, concomitant laparoscopy was not significantly associated with pregnancy (aOR, 1.27; 95% CI, 0.72–2.23) or live birth (aOR, 0.82; 95% CI, 0.47–1.44). Time to first pregnancy was also similar between groups (adjusted hazard ratio, 1.03; 95% CI, 0.75–1.41). U2b morphology, compared with U2a morphology, was not independently associated with pregnancy or live birth. Septum depth was not consistently associated with reproductive outcomes.

**Conclusion:**

No statistically significant association was detected between concomitant laparoscopy and postoperative pregnancy, live birth, miscarriage, or time to first pregnancy. Laparoscopy should be selected according to pelvic indications, anatomical complexity, and intraoperative safety requirements rather than routinely used to improve reproductive outcomes.

## Background

Septate uterus is one of the most common congenital uterine anomalies and may be associated with adverse reproductive outcomes, including infertility, recurrent pregnancy loss, preterm birth, and fetal malpresentation (1–4). Hysteroscopic septum resection allows the septum to be incised under direct visualization and restores the configuration of the uterine cavity; it is currently a commonly used surgical approach (5–7). Some centers perform concomitant laparoscopic examination or monitoring during hysteroscopic surgery to visualize the external uterine contour, assess the depth of the fundal incision, identify coexisting pelvic abnormalities, and reduce surgical risks such as uterine perforation.

With advances in three-dimensional ultrasonography, magnetic resonance imaging, and hysteroscopic techniques, the anatomy of most septate uteri can be accurately assessed before surgery (8–12). For patients without a definite indication for pelvic surgery, whether routine concomitant laparoscopy further improves perioperative safety or postoperative reproductive outcomes remains unclear (7, 13). Concomitant laparoscopy may be preferentially selected for patients with complex anatomy, an uncertain preoperative diagnosis, or suspected coexisting pelvic abnormalities; therefore, direct comparisons between the two surgical approaches may be affected by confounding by indication.

Furthermore, septate uterus represents a spectrum rather than a single homogeneous anatomical abnormality. The European Society of Human Reproduction and Embryology and European Society for Gynaecological Endoscopy classification system categorizes septate uterus as partial septate uterus, class U2a, or complete septate uterus, class U2b (14). The type and depth of the septum may more directly reflect the severity of the uterine anatomical abnormality than the use of concomitant laparoscopy and may influence postoperative pregnancy and live birth (15, 16). Therefore, this study compared the perioperative characteristics and postoperative reproductive outcomes of hysteroscopic septum resection performed alone or with concomitant laparoscopy and further examined the associations of the ESHRE/ESGE classification and septum depth with reproductive outcomes.

## Methods

### Study design and participants

This single-center retrospective cohort study included patients who underwent hysteroscopic septum resection at Chengdu Women's and Children's Central Hospital between 2017 and 2024. The study protocol was approved by the institutional ethics committee, approval number 2022–207, and registered in the Chinese Clinical Trial Registry, registration number ChiCTR2200059282.

The inclusion criteria were a diagnosis of septate uterus supported by preoperative imaging and hysteroscopic examination, treatment with hysteroscopic septum resection, and the availability of evaluable perioperative and postoperative follow-up data. The exclusion criteria were failure to undergo hysteroscopic septum resection, incomplete perioperative data, unavailable postoperative follow-up data, and concomitant reproductive procedures that could independently affect reproductive outcomes. A total of 512 patients were initially identified. Of these, 86 did not undergo hysteroscopic septum resection, 29 had incomplete perioperative data, 25 had no postoperative follow-up data, and 11 underwent other concomitant reproductive procedures. The final cohort comprised 361 patients, including 241 who underwent hysteroscopic septum resection alone and 120 who underwent hysteroscopic septum resection with concomitant laparoscopic examination or monitoring.

### Surgical procedures

All patients underwent hysteroscopic septum resection under anesthesia. A 30° rigid hysteroscope was used to evaluate the uterine cavity, both tubal ostia, and the extent of the septum. When necessary, 6 U of pituitrin was administered locally. The septum was progressively incised along its midline until the uterine cavity had been restored and both tubal ostia were located at approximately the same level. The endpoint of the incision was determined according to the fundal thickness and intraoperative findings.

In the concomitant laparoscopy group, pneumoperitoneum was established during the hysteroscopic procedure and maintained at approximately 14 mmHg. Laparoscopy was used to visualize the external uterine contour, monitor the fundal incision, assist in determining the depth of the procedure, and examine the pelvis. The decision to perform concomitant laparoscopy was made by the treating physician according to preoperative imaging, the clarity of the uterine anatomy, suspected coexisting pelvic abnormalities, previous medical history, and intraoperative safety considerations. Patients who underwent other concomitant reproductive procedures that could affect reproductive outcomes were excluded. The use of an intrauterine balloon, intrauterine device, or other intrauterine packing and the administration of hormonal or anti-adhesion treatment after surgery were determined by the surgeon according to the uterine cavity wound, bleeding, adhesion risk, patient characteristics, and clinical practice.

### Anatomical assessment

The uterine septum was retrospectively assessed using preoperative ultrasonographic or other imaging reports and hysteroscopic operative records. According to the ESHRE/ESGE classification system (14), the patients were categorized as having a partial septate uterus, class U2a, or a complete septate uterus, class U2b. Only septum-depth measurements that were explicitly documented in the examination reports and could be interpreted consistently were included in the quantitative analyses. As our institution is a tertiary regional referral center, some patients had undergone preoperative ultrasonography at other hospitals. The imaging planes, measurement methods, and reporting practices were not completely consistent across institutions, and some original images and hysteroscopic videos were unavailable. Therefore, only consistently documented information that could be reliably interpreted was reassessed. Uterine length, cavity length, and other measurements were not converted directly into septum depth.

For the exploratory analysis, patients with U2a uteri were further divided according to empirical tertiles of septum depth within the study sample: a shallower group of ≤12.3 mm, an intermediate group of >12.3–17.7 mm, and a deeper group of >17.7 mm. These thresholds were used to produce exploratory groups with relatively balanced sample sizes.

### Data collection and outcome definitions

Baseline variables included age, body mass index, previous pregnancies, previous live births, history of pregnancy loss, recurrent pregnancy loss, infertility, coexisting genital tract anomalies, surgical indication, year of surgery, ESHRE/ESGE classification, and septum depth. Perioperative and postoperative variables included intraoperative blood loss, perioperative complications, postoperative medications, the use of an intrauterine balloon or intrauterine device, second-look hysteroscopy, and postoperative menstrual changes.

The primary reproductive outcomes were at least one postoperative pregnancy and at least one postoperative live birth. Other outcomes included live birth among women who became pregnant, spontaneous or missed miscarriage, induced abortion, threatened miscarriage, preterm birth, term birth, pregnancy complications, cesarean delivery, postoperative assisted reproductive treatment, and the time from surgery to the first postoperative pregnancy. Follow-up duration was calculated from the date of surgery to the date of the last follow-up.

### Statistical analysis

Continuous variables were presented as medians and interquartile ranges after assessment of their distributions and were compared using the Mann–Whitney U test. Categorical variables were presented as numbers and percentages and were compared using the Pearson chi-square test or Fisher exact test. Logistic regression was used to evaluate the associations of concomitant laparoscopy with postoperative pregnancy, live birth, live birth among women who became pregnant, and spontaneous or missed miscarriage. The main multivariable models were adjusted for age, body mass index, gravidity, number of previous spontaneous or missed miscarriages, infertility, grouped surgical indication, year of surgery, follow-up duration, and ESHRE/ESGE classification. In the septum-depth models, ESHRE/ESGE classification was replaced by septum depth per 5-mm increase, and only patients with complete septum-depth data were included.

Kaplan–Meier analysis was used to compare the cumulative incidence of the first postoperative pregnancy between the groups, and differences were assessed using the log-rank test. Cox proportional hazards regression was further performed with adjustment for age, body mass index, gravidity, number of previous spontaneous or missed miscarriages, infertility, surgical indication, year of surgery, and ESHRE/ESGE classification. Multivariable logistic regression was also used to examine the associations of U2b versus U2a classification and each 5-mm increase in septum depth with postoperative pregnancy and live birth. All tests were two-sided, and *P* < 0.05 was considered statistically significant.

## Results

### Study population and baseline characteristics

The final analysis included 361 patients, comprising 241 in the hysteroscopy-alone group and 120 in the concomitant laparoscopy group. The median age was 29.0 years in both groups. There were no statistically significant differences between the groups in body mass index, gravidity, previous live births, number of previous spontaneous or missed miscarriages, infertility, recurrent pregnancy loss, coexisting genital tract anomalies, or year of surgery (Figure 1; Table 1).

**Figure 1:**
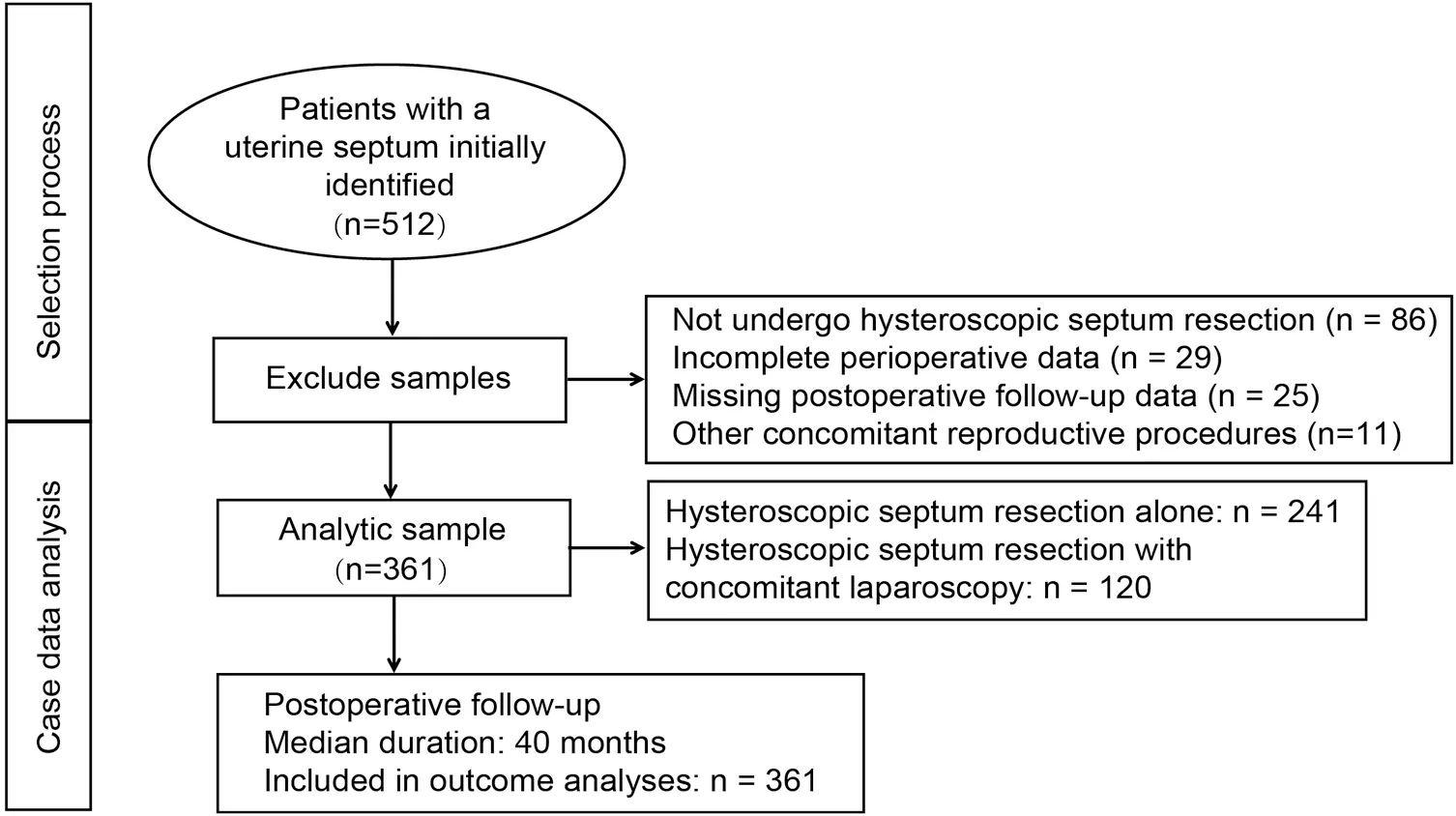
Study flow diagram.

**Table 1:** Baseline characteristics according to surgical approach.

Characteristic	Hysteroscopy alone	Hysteroscopy with laparoscopy	*P* value
Age, years	29.0 (26.0–32.0)	29.0 (26.8–32.2)	0.650^a^
Body mass index, kg/m^2^	21.2 (19.6–22.9)	21.5 (19.9–23.4)	0.309^a^
Previous pregnancies, *n*	2.0 (1.0–3.0)	1.0 (1.0–2.2)	0.081^a^
Previous live births, *n*	0.0 (0.0–1.0)	0.0 (0.0–1.0)	0.902^a^
Previous pregnancy losses, *n*	1.0 (0.0–2.0)	1.0 (0.0–2.0)	0.055^a^
Previous spontaneous/missed miscarriages, *n*	0.0 (0.0–1.0)	0.0 (0.0–1.0)	0.166^a^
History of pregnancy loss	178 (73.9)	73 (60.8)	0.011^b^
Recurrent pregnancy loss	34 (14.1)	17 (14.2)	0.988^b^
History of infertility	19 (7.9)	8 (6.7)	0.679^b^
Complete uterine septum, U2b	35 (14.5)	19 (15.8)	0.742^b^
Septum depth, mm	15.5 (11.3–21.2)	16.7 (11.6–24.0)	0.218^a^
Septum depth available	199 (82.6)	71 (59.2)	<0.001^b^
Calendar year of surgery	2021 (2019–2023)	2022 (2020–2023)	0.072^a^
Follow-up duration, months	40.0 (24.0–60.0)	39.0 (26.5–60.0)	0.668^a^

a Average and standard deviation. Mann–Whitney U.

b Number (percentage). Chi-squared Test.

The proportion of patients with a history of pregnancy loss was higher in the hysteroscopy-alone group than in the concomitant laparoscopy group, at 73.9% and 60.8%, respectively, *P* = 0.011. The median follow-up durations were 40.0 months, interquartile range 24.0–60.0 months, and 39.0 months, interquartile range 26.5–60.0 months, respectively, *P* = 0.668. The most common surgical indication in the hysteroscopy-alone group was an abnormal examination or imaging finding, accounting for 51.9%, followed by pregnancy-related indications, accounting for 37.3%. In the concomitant laparoscopy group, pregnancy-related indications, abnormal examination or imaging findings, and abnormal uterine bleeding or endometrial polyps accounted for 34.2%, 30.8%, and 27.5%, respectively (Table 1).

### Uterine septum classification and septum depth

Among the 361 patients, 307 were classified as U2a and 54 as U2b. The proportions of patients with U2b uteri were 14.5% in the hysteroscopy-alone group and 15.8% in the concomitant laparoscopy group, *P* = 0.742 (Table 1). Septum-depth measurements were available for 270 patients, corresponding to an overall reporting rate of 74.8%. Septum-depth measurements were available for 241 patients with U2a uteri, a reporting rate of 78.5%, and 29 patients with U2b uteri, a reporting rate of 53.7%. Septum depth was significantly greater in patients with U2b uteri than in those with U2a uteri, with median values of 29.6 mm, interquartile range 23.5–37.1 mm, and 15.0 mm, interquartile range 11.0–19.1 mm, respectively, *P* < 0.001.

Among patients with available measurements, the median septum depths in the hysteroscopy-alone and concomitant laparoscopy groups were 15.5 mm, interquartile range 11.3–21.2 mm, and 16.7 mm, interquartile range 11.6–24.0 mm, respectively, *P* = 0.218. However, the corresponding septum-depth reporting rates were 82.6% and 59.2%, respectively, *P* < 0.001 (Table 1).

### Perioperative and postoperative management

The median intraoperative blood loss was 10.0 mL in both groups, *P* = 0.271. Two complications were recorded in the hysteroscopy-alone group, both involving bleeding, and one complication was recorded in the concomitant laparoscopy group, involving fever (Table 2). The complication rate was 0.8% in each group, *P* = 1.000. The overall distribution of postoperative medications did not differ significantly between the groups, *P* = 0.151. Intrauterine balloon use was more frequent in the hysteroscopy-alone group than in the concomitant laparoscopy group, at 57.3% and 19.2%, respectively, *P* < 0.001. The rates of second-look hysteroscopy and postoperative menstrual patterns did not differ significantly between the groups.

**Table 2:** Perioperative and postoperative characteristics according to surgical approach.

Characteristic	Hysteroscopy alone	Hysteroscopy with laparoscopy	*P* value
Intraoperative blood loss, mL	10.0 (10.0–20.0)	10.0 (10.0–20.0)	0.271^a^
Intraoperative complication	0 (0.0)	0 (0.0)	1.000^b^
Postoperative medication, overall			0.151^c^
None	83 (34.4)	40 (33.3)	
Hormonal therapy	72 (29.9)	40 (33.3)	
Anti-adhesion agent	42 (17.4)	28 (23.3)	
Hormonal therapy plus anti-adhesion agent	44 (18.3)	12 (10.0)	
Intrauterine device/packing, overall			<0.001^c^
None	89 (36.9)	85 (70.8)	
Intrauterine device	14 (5.8)	12 (10.0)	
Balloon	138 (57.3)	23 (19.2)	
Second-look hysteroscopy	21 (8.7)	6 (5.0)	0.206^c^
Postoperative menstrual pattern, overall			0.546^b^
Normal	198 (82.2)	104 (86.7)	
Decreased flow	37 (15.4)	14 (11.7)	
Increased flow	6 (2.5)	2 (1.7)	

a Average and standard deviation. Mann–Whitney U.

b Number (percentage). Fisher Exact Test.

c Number (percentage). Chi-squared Test.

### Postoperative reproductive outcomes

Postoperative pregnancy occurred in 137 patients in the hysteroscopy-alone group and 73 in the concomitant laparoscopy group, corresponding to pregnancy rates of 56.8% and 60.8%, respectively, *P* = 0.469. At least one live birth occurred in 103 and 47 patients, corresponding to overall live birth rates of 42.7% and 39.2%, respectively, *P* = 0.517 (Table 3). Among women who became pregnant, the live birth rates were 75.2% and 64.4%, respectively, *P* = 0.099; the rates of spontaneous or missed miscarriage were 15.3% and 23.3%, respectively, *P* = 0.154; and the rates of any pregnancy loss were 25.5% and 28.8%, respectively, *P* = 0.615. No statistically significant differences were observed in postoperative assisted reproductive treatment, induced abortion, preterm birth, term birth, pregnancy complications, or cesarean delivery.

**Table 3:** Postoperative reproductive and obstetric outcomes according to surgical approach.

Outcome	Population	Hysteroscopy alone	Hysteroscopy with laparoscopy	*P* value
At least one postoperative Pregnancy	All	137 (56.8)	73 (60.8)	0.469
Postoperative use of assisted reproduction	All	11 (4.6)	5 (4.2)	0.863
Time from surgery to Pregnancy, months	All	9.0 (5.0–14.5)	12.0 (6.0–18.0)	0.197
At least one Live birth, All women	All	103 (42.7)	47 (39.2)	0.517
At least one Pregnancy loss	Preg	35 (25.5)	21 (28.8)	0.615
At least one induced abortion	Preg	15 (10.9)	6 (8.2)	0.530
At least one spontaneous/missed miscarriage	Preg	21 (15.3)	17 (23.3)	0.154
Threatened miscarriage	Preg	52 (38.0)	17 (23.3)	0.031
At least one Live birth among Pregnant women	Preg	103 (75.2)	47 (64.4)	0.099
At least one preterm birth	Live	12 (11.7)	5 (10.6)	0.856
At least one term birth	Live	90 (87.4)	41 (87.2)	0.980
Any Pregnancy complication	Live	33 (32.0)	18 (38.3)	0.453
Cesarean deLivery	Live	74 (71.8)	32 (68.1)	0.639

The rates of threatened miscarriage were 38.0% in the hysteroscopy-alone group and 23.3% in the concomitant laparoscopy group, with an unadjusted *P* value of 0.031. After false-discovery-rate correction for the secondary outcomes, the adjusted *P* value was 0.311 and was no longer statistically significant (Table 3).

### Association between concomitant laparoscopy and reproductive outcomes

In the unadjusted analysis, concomitant laparoscopy was not significantly associated with postoperative pregnancy, with an odds ratio of 1.18, 95% confidence interval 0.75–1.84, *P* = 0.470 (Table 4; Figure 2). After adjustment for the ESHRE/ESGE classification and other confounders, the adjusted odds ratio was 1.27, 95% confidence interval 0.72–2.23, *P* = 0.406. In the complete-case septum-depth model, the adjusted odds ratio was 1.38, 95% confidence interval 0.67–2.85, *P* = 0.380. Concomitant laparoscopy was not significantly associated with overall postoperative live birth. The unadjusted odds ratio was 0.86, 95% confidence interval 0.55–1.35, *P* = 0.517. The adjusted odds ratio was 0.82, 95% confidence interval 0.47–1.44, *P* = 0.495, in the ESHRE/ESGE classification model and 0.74, 95% confidence interval 0.37–1.46, *P* = 0.379, in the complete-case septum-depth model.

**Table 4:** Associations between concomitant laparoscopy and postoperative reproductive outcomes.

Outcome	Model	*N*	Events	OR (95% CI)	*P* value
Postoperative pregnancy	Crude	361	210	1.18 (0.75–1.84)	0.470
	Adjusted, ESHRE/ESGE class	361	210	1.27 (0.72–2.23)	0.406
	Adjusted, septum depth	270	170	1.38 (0.67–2.85)	0.380
Postoperative live birth	Crude	361	150	0.86 (0.55–1.35)	0.517
	Adjusted, ESHRE/ESGE class	361	150	0.82 (0.47–1.44)	0.495
	Adjusted, septum depth	270	129	0.74 (0.37–1.46)	0.379
Live birth among pregnant women	Crude	210	150	0.60 (0.32–1.11)	0.101
	Adjusted, ESHRE/ESGE class	210	150	0.44 (0.20–1.00)	0.051
	Adjusted, septum depth	170	129	0.29 (0.11–0.82)	0.019
Spontaneous/missed miscarriage among pregnant women	Crude	210	38	1.68 (0.82–3.43)	0.156
	Adjusted, ESHRE/ESGE class	210	38	1.52 (0.66–3.50)	0.327
	Adjusted, septum depth	170	29	1.61 (0.59–4.39)	0.350

**Figure 2:**
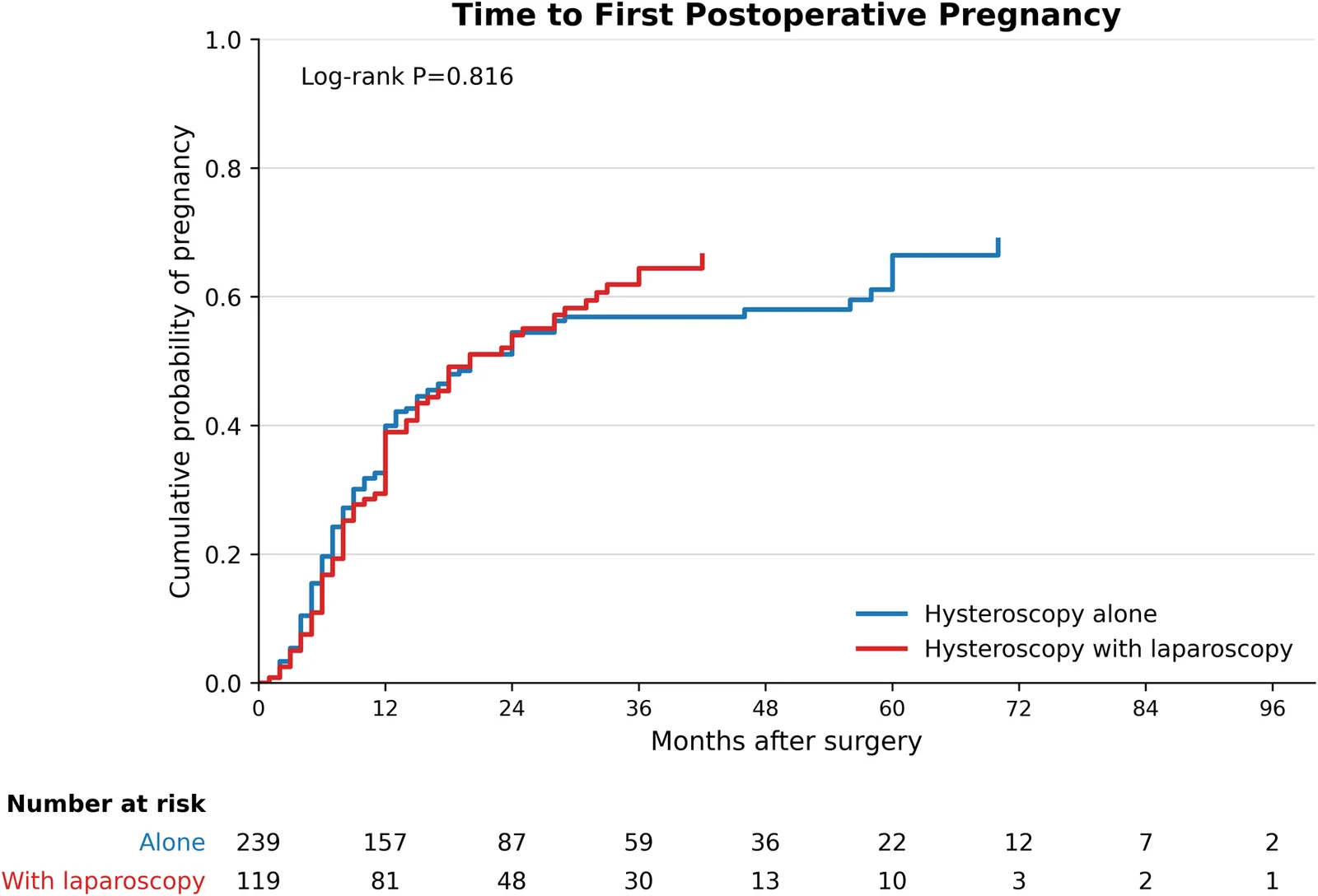
Association of concomitant laparoscopy with postoperative reproductive outcomes. Forest plots show crude and adjusted odds ratios with 95% confidence intervals for postoperative pregnancy, overall live birth, live birth among women who became pregnant, and spontaneous or missed miscarriage. Concomitant laparoscopy was not significantly associated with these outcomes in the crude or ESHRE/ESGE classification-adjusted models. In the complete-case septum-depth model, concomitant laparoscopy was associated with a lower likelihood of live birth among women who became pregnant (adjusted OR, 0.29; 95% CI, 0.11–0.82; *P* = 0.019), although this estimate may have been affected by selection bias resulting from missing septum-depth data. The vertical dashed line indicates an OR of 1.0.

Among women who became pregnant, the unadjusted odds ratio for live birth associated with concomitant laparoscopy was 0.60, 95% confidence interval 0.32–1.11, *P* = 0.101. The adjusted odds ratio was 0.44, 95% confidence interval 0.20–1.00, *P* = 0.051, in the ESHRE/ESGE classification model. In the complete-case septum-depth model, the adjusted odds ratio was 0.29, 95% confidence interval 0.11–0.82, *P* = 0.019. This model included only 170 women who became pregnant and had complete septum-depth data. Because missing septum-depth data were significantly associated with both surgical approach and reproductive outcomes, this estimate may have been affected by selection bias (Table 4; Figure 2). Concomitant laparoscopy was not significantly associated with spontaneous or missed miscarriage among women who became pregnant. The unadjusted odds ratio was 1.68, 95% confidence interval 0.82–3.43, *P* = 0.156. The adjusted odds ratios were 1.52, 95% confidence interval 0.66–3.50, *P* = 0.327, in the ESHRE/ESGE classification model and 1.61, 95% confidence interval 0.59–4.39, *P* = 0.350, in the complete-case septum-depth model.

### Time to first postoperative pregnancy

The median times from surgery to the first pregnancy were 9.0 months, interquartile range 5.0–14.5 months, in the hysteroscopy-alone group and 12.0 months, interquartile range 6.0–18.0 months, in the concomitant laparoscopy group, *P* = 0.197 (Figure 3). Kaplan–Meier analysis showed no significant difference in the time to first postoperative pregnancy between the groups, log-rank *P* = 0.816. After adjustment for the relevant confounders, concomitant laparoscopy was not significantly associated with the time to first postoperative pregnancy, with an adjusted hazard ratio of 1.03, 95% confidence interval 0.75–1.41, *P* = 0.872. In the complete-case septum-depth model, the adjusted hazard ratio was 0.98, 95% confidence interval 0.68–1.42, *P* = 0.923.

**Figure 3:**
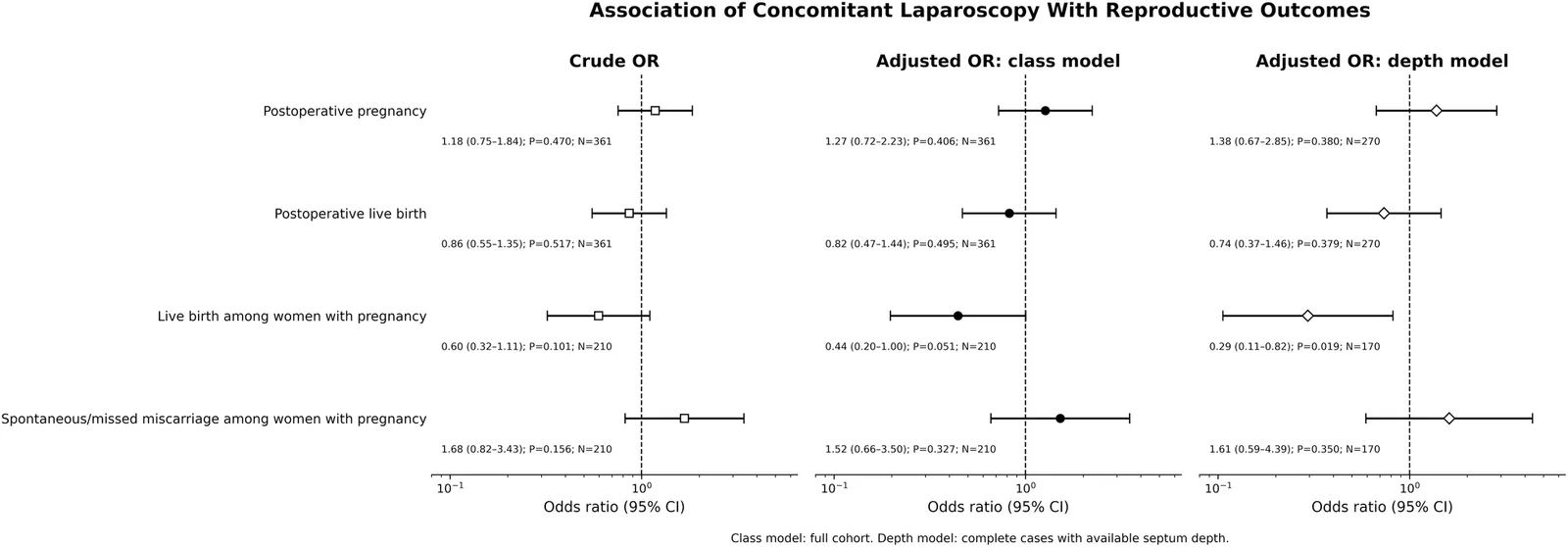
Time to first postoperative pregnancy according to surgical approach. Kaplan–Meier curves compare the cumulative probability of first postoperative pregnancy between the hysteroscopy-alone and concomitant-laparoscopy groups. No significant difference was observed between the groups (log-rank *P* = 0.816). After adjustment for relevant confounders, concomitant laparoscopy was not significantly associated with time to first postoperative pregnancy (adjusted HR, 1.03; 95% CI, 0.75–1.41; *P* = 0.872). The numbers of patients at risk are shown below the plot.

### Anatomical characteristics and reproductive outcomes

After adjustment for operative approach and other confounders, U2b versus U2a morphology was not significantly associated with postoperative pregnancy (adjusted OR, 1.24; 95% CI, 0.62–2.47; *P* = 0.549) or live birth (adjusted OR, 0.84; 95% CI, 0.41–1.72; *P* = 0.634) Each 5-mm increase in septum depth was not independently associated with postoperative pregnancy, adjusted odds ratio 0.90, 95% confidence interval 0.76–1.05, *P* = 0.178, or postoperative live birth, adjusted odds ratio 0.88, 95% confidence interval 0.74–1.03, *P* = 0.110 (Table 5). Among the 241 patients with U2a uteri and available septum-depth measurements, the shallower, intermediate, and deeper groups included 81, 80, and 80 patients, respectively. Their postoperative pregnancy rates were 65.4%, 66.2%, and 52.5%, respectively, and their live birth rates were 55.6%, 42.5%, and 38.8%, respectively.

**Table 5:** Associations of ESHRE/ESGE classification and septum depth with postoperative pregnancy and live birth.

Analysis	Outcome	*N*	Events	Adjusted OR (95% CI)	*P* value
U2b versus U2a	Postoperative pregnancy	361	210	1.24 (0.62–2.47)	0.549
Septum depth per 5-mm increase	Postoperative pregnancy	270	170	0.90 (0.76–1.05)	0.178
U2b versus U2a	Postoperative live birth	361	150	0.84 (0.41–1.72)	0.634
Septum depth per 5-mm increase	Postoperative live birth	270	129	0.88 (0.74–1.03)	0.110
Middle versus lower	Postoperative pregnancy	241	148	0.93 (0.44–1.97)	0.848
Upper versus lower	Postoperative pregnancy	241	148	0.54 (0.26–1.14)	0.108
Continuous depth within U2a, per 5 mm	Postoperative pregnancy	241	148	0.85 (0.68–1.06)	0.145
Middle versus lower	Postoperative live birth	241	110	0.50 (0.24–1.03)	0.061
Upper versus lower	Postoperative live birth	241	110	0.36 (0.17–0.77)	0.009
Continuous depth within U2a, per 5 mm	Postoperative live birth	241	110	0.77 (0.61–0.97)	0.025

ESHRE, European Society of Human Reproduction and Embryology; ESGE, European Society for Gynaecological Endoscopy; FDR, false discovery rate; IQR, interquartile range; OR, odds ratio.

After adjustment for the relevant confounders, the adjusted odds ratios for postoperative pregnancy were 0.93, 95% confidence interval 0.44–1.97, *P* = 0.848, for the intermediate versus shallower group and 0.54, 95% confidence interval 0.26–1.14, *P* = 0.108, for the deeper versus shallower group (Table 5). The adjusted odds ratios for postoperative live birth were 0.50, 95% confidence interval 0.24–1.03, *P* = 0.061, and 0.36, 95% confidence interval 0.17–0.77, *P* = 0.009, respectively. After false-discovery-rate correction, the corresponding *P* values were 0.121 and 0.052. Among patients with U2a uteri, each 5-mm increase in septum depth was associated with an adjusted odds ratio of 0.85 for postoperative pregnancy, 95% confidence interval 0.68–1.06, unadjusted *P* = 0.145 and false-discovery-rate-adjusted *P* = 0.174. The adjusted odds ratio for postoperative live birth was 0.77, 95% confidence interval 0.61–0.97, unadjusted *P* = 0.025 and false-discovery-rate-adjusted *P* = 0.074.

### Sensitivity analyses

After restricting the analyses to patients with U2a uteri, those with at least 24 months of follow-up, those with shared surgical indications, those without coexisting genital tract anomalies, and those who did not receive postoperative assisted reproductive treatment, concomitant laparoscopy remained unassociated with postoperative pregnancy or live birth.

In the stabilized inverse-probability-weighted analysis, the odds ratios associated with concomitant laparoscopy were 1.21, 95% confidence interval 0.74–2.01, *P* = 0.448, for postoperative pregnancy and 1.03, 95% confidence interval 0.61–1.77, *P* = 0.902, for postoperative live birth. The sensitivity analyses were generally consistent with the main analyses.

## Discussion

This study compared reproductive outcomes after hysteroscopic septum resection performed alone or with concomitant laparoscopy and further evaluated the roles of the ESHRE/ESGE classification and septum depth. After adjustment for baseline characteristics, surgical indication, year of surgery, follow-up duration, and septum classification, no statistically significant association was detected between concomitant laparoscopy and postoperative pregnancy or overall live birth. These findings are consistent with the broader evidence showing uncertainty regarding the extent to which additional surgical intervention improves reproductive outcomes after correction of a septate uterus (8, 17, 18). Kaplan–Meier and Cox regression analyses also showed no significant difference in the time to first postoperative pregnancy between the groups. Propensity-score weighting and multiple sensitivity analyses yielded findings in the same direction as the main analyses (9, 19–21).

Laparoscopy provides an external view of the uterus during hysteroscopic septum resection, assists in assessing the external uterine contour and depth of the fundal incision, and may identify pelvic adhesions, endometriosis, or other pelvic abnormalities. For patients with an uncertain preoperative diagnosis, complex anatomy, or a specific indication for pelvic surgery, laparoscopy may retain diagnostic and safety-monitoring value (7, 13). The present findings support individualized selection of concomitant laparoscopy according to pelvic assessment, anatomical complexity, and intraoperative safety requirements rather than its routine use solely to increase postoperative pregnancy or live birth rates.

The choice of concomitant laparoscopy was not randomized but was determined by clinicians according to the patient's condition. The two groups differed in surgical indications and postoperative intrauterine management, indicating that surgical approach was influenced by case characteristics, surgeon judgment, and changes in clinical practice. The main models adjusted for surgical indication, year of surgery, and septum classification, and the analyses restricted to shared surgical indications and the stabilized inverse-probability-weighted analyses similarly detected no statistically significant association between concomitant laparoscopy and the primary reproductive outcomes. Nevertheless, the retrospective design could not fully account for incompletely documented endometriosis, pelvic adhesions, tubal status, or anatomical complexity, and confounding by indication cannot be excluded.

No statistically significant differences in postoperative pregnancy or live birth were detected between patients with U2a and U2b uteri. Although patients with U2b uteri had substantially deeper septa, the preoperative septum classification alone may be insufficient to predict reproductive outcomes after hysteroscopic correction. Previous studies of complete and partial septa have also produced heterogeneous findings regarding the effects of anatomical subtype and septum size on postoperative reproductive outcomes (22–24). Age, ovarian reserve, embryo quality, male factors, tubal status, previous pregnancy history, and postoperative assisted reproductive treatment may collectively determine the final reproductive outcome.

Among patients with U2a uteri, the postoperative live birth rate tended to decrease as septum depth increased. The adjusted odds ratio for live birth was 0.36 for the deeper group versus the shallower group, and the adjusted odds ratio was 0.77 for each 5-mm increase in septum depth. However, neither association remained statistically significant after false-discovery-rate correction. These findings suggest that septum depth may have potential prognostic value but should currently be regarded only as an exploratory signal. The empirical tertile thresholds were derived from the present sample and have not been externally validated; therefore, they should not be directly used for clinical risk stratification or to determine surgical indications.

The effects of hysteroscopic septum resection may not be limited to mechanical restoration of the uterine cavity. Studies of hysteroscopic endometrial fundal incision have suggested that controlled fundal incision may influence endometrial receptivity through local tissue repair, the release of inflammatory mediators, angiogenesis, and endometrial remodeling (25–27). This mechanism may be relevant to implantation and pregnancy after surgery in some patients with minor fundal abnormalities. However, the present study did not include a nonsurgical control group or assess endometrial receptivity or local biological markers. It was therefore not possible to determine whether postoperative reproductive outcomes resulted from anatomical correction, the potential biological effects of fundal incision, or other clinical factors.

Selective missingness of septum-depth data was an important limitation of the anatomical analyses. Some ultrasonographic reports for patients with U2b uteri included complete uterine sections and uterine length but did not separately report septum depth. To preserve statistical rigor, uterine length was not treated as septum depth, resulting in a lower septum-depth reporting rate among patients with U2b uteri than among those with U2a uteri.

The recorded complication rates were low in both groups, but only three events occurred in the entire cohort. The available sample therefore lacked sufficient power to compare perioperative safety accurately. The absence of a statistically significant difference should not be interpreted as evidence that the two approaches are equivalent in safety and does not exclude a potential protective role of laparoscopic monitoring in patients with complex anatomy or a higher surgical risk (7, 13).

The strengths of this study include its relatively large sample, a median follow-up duration of approximately 40 months, and the evaluation of pregnancy, live birth, pregnancy loss, and time to first pregnancy. The analyses also incorporated the ESHRE/ESGE classification and septum depth and used multivariable regression, time-to-event analysis, stabilized inverse-probability weighting, and multiple sensitivity analyses to evaluate the robustness of the findings. Multiple-testing correction was applied to the secondary outcomes and exploratory U2a analyses.

This study also has several limitations. First, its single-center retrospective design was susceptible to selection bias, information bias, and unmeasured confounding and could not establish a causal relationship between surgical approach and reproductive outcomes. Second, the choice to perform concomitant laparoscopy was based on clinical circumstances, and the groups may have differed in incompletely recorded anatomical characteristics and pelvic abnormalities. Third, because our institution is a tertiary regional referral center, some patients underwent preoperative examination at other hospitals. Differences in imaging-reporting practices resulted in selective missingness of septum-depth data and prevented independent imaging reassessment for all cases. Fourth, excluding patients without postoperative follow-up data may have introduced selection bias. The number of events for some outcomes was limited and the confidence intervals for several estimates were wide; therefore, a lack of statistical significance should not be interpreted as equivalence between the two approaches.

## Conclusion

In this retrospective cohort, no statistically significant association was detected between concomitant laparoscopy during hysteroscopic septum resection and postoperative pregnancy, overall live birth, spontaneous or missed miscarriage, or time to first pregnancy. Neither ESHRE/ESGE septum classification nor septum depth was a consistent independent prognostic factor.

The decision to perform concomitant laparoscopy should be based on definite indications for pelvic assessment or treatment, the complexity of the uterine anatomy, individual patient characteristics, and intraoperative safety requirements. Prospective multicenter studies using standardized imaging measurements are needed to further determine the optimal surgical strategy for patients with different anatomical phenotypes of septate uterus.

## Data Availability

The raw data supporting the conclusions of this article will be made available by the authors, without undue reservation.
